# Evaluating the efficacy of LDH and inflammatory indices in discriminating neonatal respiratory distress syndrome from transient tachypnea of the newborns

**DOI:** 10.1186/s12887-025-05519-6

**Published:** 2025-07-03

**Authors:** Hala M. Sakhr, Fayed HM, Amany E. Abdel Aziz, Heba M. Qubaisy

**Affiliations:** 1 Department of Pediatrics, Faculty of Medicine, South Valley University, Qena, Egypt; 2 Department of Clinical and Chemical Pathology, Faculty of Medicine, South Valley University, Qena, Egypt

**Keywords:** Inflammatory indices, Respiratory distress syndrome, Transient tachypnea of the newborn

## Abstract

**Background:**

Early discrimination between transient tachypnea of the newborn (TTN) and respiratory distress syndrome (RDS) is critical for establishing timely targeted therapies. Our aim was to assess the efficacy of LDH, platelet indices, and systemic inflammatory indices in distinguishing neonatal RDS from TTN early.

**Methods:**

In total, 300 neonates were enrolled in this case-control study. Lactate dehydrogenase (LDH) levels were estimated. Platelet and systemic inflammatory indices were calculated using complete blood count.

**Results:**

The RDS group exhibited substantially higher serum levels of LDH, neutrophil-lymphocyte ratio (NLR), and Systemic immune-inflammation index (SII) than the TTN and control groups. Platelet-lymphocyte ratio (PLR) and monocyte-lymphocyte ratio (MLR (were significantly higher in the RDS group than in the TTN group. The RDS group also had the lowest median platelet count, platelet mass index, and WBCs/ mean platelet volume (MPV), but much higher MPV/platelet count than the TTN and control groups. The TTN group had more WBCs, lymphocyte percentage, and count, but a lower neutrophil percentage than the RDS group. The ROC curve study demonstrated that serum LDH at a cut-off level of > 660 U/L can discriminate between the RDS and TTN groups. NLR had the highest sensitivity, PLR had the highest specificity, and SII at a cut-off level > 245.57, had 67% sensitivity and 56% specificity. Significant positive correlations were detected between Downe score with NLR (*r* = 0.317**, *p* = 0.001), PLR (*r* = 0.261**, *p* = 0.009), and SII (*r* = 0.270**, *p* = 0.007).

**Conclusion:**

LDH levels, platelets, and systematic inflammatory indices could serve as affordable biomarkers for the early distinction between RDS and TTN.

## Introduction

Transient tachypnea of the newborn (TTN) is a parenchymal lung condition that causes pulmonary oedema due to delayed resorption and elimination of fetal alveolar fluid. It is the most prevalent cause of respiratory distress in late preterm and term newborns [1]. TTN cases have low oxygen content; therefore, lower energy is provided by glucose metabolism via the anaerobic pathway, and pyruvate is deoxidized into lactate-by-lactate dehydrogenase [2].

Respiratory distress syndrome (RDS) typically appears within 24 h of delivery and worsens within the first 48 h [3]. Lung immaturity, both physiologically and anatomically, causes RDS. Inadequate amounts of pulmonary surfactants impair alveolar integrity and proper gas exchange, leading to atelectasis and impaired lung compliance [4].

The pathogenesis of neonatal RDS is complex and multifactorial, causing inflammatory damage to the immature lungs. Secondary insults such as traumatic stabilization procedures and mechanical ventilation can prolong and potentially exacerbate the inflammatory process [5].

Lactate dehydrogenase (LDH) is an intracellular enzyme found in almost all human tissues. Injured cells that have lost cell membrane integrity leak LDH into extracellular regions [6]. Evaluating serum LDH levels provides insights into tissue damage and stress, and plasma LDH is also an indicator of body tissue hypoxia. Previous studies on LDH levels in neonates have found a correlation between LDH concentration and asphyxia [7–9].

In recent years, there has been growing interest in the use of common haematological markers, including systemic inflammatory indices generated from whole blood count values. Very little evidence is available on systemic inflammatory indicators in newborns [10–11]. Cakir et al. [12] found that a low systemic immune-inflammation index value in extremely low-birth-weight preterm infants could be a unique predictor of mortality.

Platelets (PLTs) maintain endothelial barrier function by releasing soluble compounds, blocking endothelial barrier gaps, preserving cell structure, and promoting endothelial cell proliferation [13]. There is strong evidence that PLTs can boost endothelial function and reduce permeability. Appropriate levels of circulating PLTs may be crucial in maintaining the integrity of the alveolar-capillary barrier and other capillary beds [14]. Platelet mass provides a more accurate reflection of platelet function than does platelet count alone [15].

Early discrimination between RDS and TTN is critical for early diagnosis and a better prognosis; nevertheless, both disorders are highly frequent in terms of incidence and clinical expression, but have fundamentally different therapy approaches [16–17]. This study sought to determine the diagnostic significance of LDH, platelet indices, and inflammatory indices in separating neonatal respiratory distress syndrome from transient tachypnea in neonates.

## Patient and methods

### Research design

An observational case-control study involving 300 newborns aged ≥ 28 weeks. 200 cases (divided into two subgroups; 100 cases diagnosed with RDS and 100 cases diagnosed with TTN) compared with 100 healthy newborns as a control group. Cases are diagnosed as having RDS based on clinical features such as grunting, retractions, nasal flaring, cyanosis, tachypnea, need for oxygen or pressure support before intubation, and radiographic findings such as a diffuse reticular-granular pattern, ground-glass appearance, and superimposed air bronchogram [18]. TTN cases are diagnosed based on initial clinical signs, including tachypnea within the first 6 h postnatally, persistence for at least 12 h, and radiologic findings such as lung hyperinflation, prominent pulmonary vascular markings, diaphragm flattening, or fluid in fissures [2]. The study was conducted with newborns hospitalized in the Neonatal Intensive Care Unit (NICU) of Qena University Hospital, South Valley University, Egypt, between March 2023 and February 2024, after approval from the patients’ caregivers and the faculty’s ethical committee (ethical code number: SVUMEDPED0254245850).

The study excluded post-term cases, cases of respiratory distress due to pneumonia, meconium aspiration syndrome, congenital lung diseases, sepsis, air leak syndrome (pneumothorax), hypoxic-ischemic encephalopathy, cardiac patients, congenital diaphragmatic hernia, major congenital anomalies, and neonates requiring cardiopulmonary resuscitation at birth. The sample size was calculated with 95% confidence, 80% power, a cases-to-control ratio (2:1), a postulated odds ratio of ≥ 2, and an expected prevalence of 46.5% [19].

### Clinical assessment

Comprehensive maternal and neonatal histories were obtained. All babies underwent a complete physical examination. Vital signs, anthropometric measurements, and gestational age were determined using the Modified Ballard Score [20]. Infants were classified as appropriate for gestational age (AGA) if their weight fell between the 10th and 90th percentiles of the average birth weight for the same gestational age; small for gestational age (SGA) if their weight fell below the 10th percentile; and large for gestational age (LGA) if their weight fell above the 90th percentile [21]. Pulse oximetry (BLT M9000 monitor) was used to assess all neonate’s oxygen percentages. The Downes score is used to assess distress levels at admission using 5 parameters; respiratory rate, retractions, cyanosis, air entry, and grunting. A score of zero indicates no distress, a score of 1–4 mild RD, a score of 5–7 moderate RD, and a score of > 7 indicates severe distress or impending respiratory failure) [22].

### Laboratory evaluation

Blood samples were collected at admission time within 24 h postpartum. Four ml of venous blood was collected under complete aseptic conditions; 2 ml of blood was added to an EDTA tube for a complete blood count (CBC) with differential (cross-referenced with normal reference ranges for Neonates [23], by using cell dyne-Ruby (Abbott Diagnostics-Santa Clara- California-USA), automated cell counter and in accordance with the manufacturer’s instructions, background checks, and machine maintenance were carried out to maintain the complete blood count analyzer’s quality for commercially manufactured known blood samples (normal, low, and high). The other 2 ml of blood was added to a plain tube and left to clot at room temperature for 10–20 min; the tube was then centrifuged for 20 min at 2000–3000 rpm; and serum was collected for the estimation of lactate dehydrogenase (LDH) (Beckman Coulter AU 480-CA-USA) where in under normal conditions, serum LDH levels in newborn are less than 580 U/L [24]. Capillary blood gas was performed (ABL800-FLEX, Radiometer, Denmark) for the RDS and TTN groups. Hypoxemia was identified as Po2 < 60 mmHg or SpO2 < 90% [25–26].

Platelet indices provided from a full blood picture include platelet count, platelet distribution width (PDW), mean platelet volume (MPV), and platelet large cell ratio (PLCR). Other indexes are computed, as follows: Platelet Mass Index (PMI) = mean platelet volume x platelet count /1000 (fL/mL) [27]. The P2/MS = [platelet count (10^9^/l)] ^2^/ [monocyte fraction (%) × segmented neutrophil fraction (%)] [28].

The systemic inflammatory indicators were computed, such as the systemic immune-inflammation index (SII) = (neutrophil count × platelet count) / lymphocyte count [29]. The platelet-lymphocyte ratio (PLR) was obtained by dividing the platelet count by the lymphocyte count [30]. The monocyte-lymphocyte ratio (MLR) is determined by dividing the monocyte count by the lymphocyte count. To calculate the neutrophil-to-lymphocyte ratio (NLR), divide the neutrophil count by the lymphocyte count [31]. The systemic inflammatory response index (SIRI) is derived as (neutrophil count x monocyte count) divided by lymphocyte count [32].

### Imaging evaluation

Two experienced radiology doctors reviewed each case after it was X-rayed in the chest using a portable IMD (Basic 4003 Raclo, Italy) to determine the radiological features of RDS and TTN and to rule out cases that were ineligible. Echocardiography (with the GE Vivid S5, USA ultrasound machine) was conducted on patients suspected of having concurrent congenital heart issues to be excluded from the study.

### Statistical analysis

Statistical Program for Social Sciences software version 24 was used. Our data were determined to be non-normally distributed, and they are provided as median and interquartile range (IQR). The two groups were compared using Mann-Whitney tests for continuous variables. Categorical variables are expressed as numbers (N) and percentages (%), with the chi-square test used to compare two groups. Spearman’s rank correlation was employed to investigate the link between non-normally distributed continuous variables. The receiver operating characteristic curve (ROC) was used to determine the cut-off values at the highest sensitivity, specificity, and positive and negative predictive values. *P*-values < 0.05 were considered statistically significant.

## Results

The current study included 300 newborns (gestational age ranging from 28 to 38 weeks): 100 with RDS, 100 with TTN, and 100 healthy neonates. Neonates with RDS had the shortest median gestational age of 31 weeks (IQR, 30–33), whereas the TTN group had a median gestational age of 33 weeks (IQR, 32–35). There were no significant differences in sex or residence among the three groups, but the TTN group had a much higher percentage of consanguineous marriages. With statistically significant differences, neonates with RDS had the highest percentage of small for gestational age cases (18%), while the TTN group had the highest percentage of large for gestational age cases (38%). Compared to TTN and control cases, RDS infants had lower temperatures, higher heart and respiratory rates, and lower oxygen saturation levels, indicating greater severity and immaturity (Table 1).

**Table 1:** Demographic and clinical data for the examined neonates

Variables		RDS (*N* = 100)	TTN (*N* = 100)	Controls (*N* = 100)	*P*-value
Gestational age (W) Median (IQR)		31 (30–33)	33 (32–35)	37 (37–38)	P1 < 0.001 * P2 < 0.001 * P3 < 0.001 *
Gender, *n*%	Males	59 (59%)	68 (68%)	56 (56%)	P1 = 0.76 P2 = 0.08 P3 = 0.19
	Females	41 (41%)	32 (32%)	44 (44%)	
Residence, *n*%	Rural	51 (51%)	49 (54%)	54 (54%)	P1 = 0.67 P2 = 0.48 P3 = 0.78
	Urban	49 (49%)	51 (51%)	46 (46%)	
Consanguinity, *n*%	Positive	16 (16%)	34 (34%)	17 (17%)	P1 = 0.85 P2 = 0.006 * P3 = 0.003 *
	Negative	84 (84%)	66 (66%)	83 (83%)	
Birth weight- for Gestational age, *n*%	SGA AGA LGA	18 (18.0%) 71 (71.0%) 11 (11.0%)	1 (1.0%) 61 (61.0%) 38 (38.0%)	0 (0.0%) 100 (100%) 0 (0.0%)	P1 < 0.001 * P2 < 0.001 * P3 < 0.001 *
Temperature (°C) Median (IQR)		36.8 (36.5–37)	37 (36.9–37)	37 (37–37)	P1 < 0.001 * P2 = 0.34 P3 < 0.001 *
Heart rate (beat/minute) Median (IQR)		131 (115–139)	130 (122–133)	119 (110–122)	P1 < 0.001 * P2 < 0.001 * P3 = 0.69
Respiratory rate (breath/minute) Median (IQR)		80 (70–88)	66 (65–70)	37 (35–38)	P1 < 0.001 * P2 < 0.001 * P3 < 0.001 *
O2 saturation Median (IQR)		86 (78–88)	93 (92–94)	99 (98–99)	P1 < 0.001 * P2 < 0.001 * P3 < 0.001 *

*: Significant; P1: RDS vs. control groups; P2: TTN vs. control groups; P3: RDS vs. TTN groups; AGA: Appropriate for gestational age; LGA: Large for gestational age; SGA: small for gestational age

We found a significantly higher median Downe score in RDS [7 (IQR, 6–8)] than in TTN [3 (IQR, 2–3)], indicating more severe respiratory distress. TTN neonates exhibited higher median pH [7.35 (7.33–7.36)], PO2 [67 (65–75)], and HCO3 levels [20 (19–21)] versus RDS cases [PH = 7.32 (7.27–7.35), PO2 = 51 (44–55), and HCO3 = 17 (14–19) respectively], indicating better oxygenation and a more favourable acid-base balance in transient tachypnea and a more acidotic state in RDS cases. RDS cases required more extensive oxygen therapy and had a longer hospital stay than TTN cases, with a less favourable prognosis and higher fatality rates (Table 2).

**Table 2:** Oxygen therapy in the evaluated patients group

		RDS (*N* = 100)	TTN (*N* = 100)	*P*-value
Downe score	Median (IQR)	7 (6–8)	3 (2–3)	< 0.001 *
Type of O2 therapy	Nasal Cannula	8 (8%)	90 (90%)	< 0.001 **
	CPAP	45 (45%)	10 (10%)	
	MV	47 (47%)	0 (0%)	
Duration of hospital admission (day)		6 (4–7)	3 (2–4)	< 0.001 *
ABG findings	pH	7.32 (7.27–7.35)	7.35 (7.33–7.36)	< 0.001 *
	PO_2_	51 (44–55)	67 (65–75)	< 0.001 *
	PCO_2_	40 (33–45)	40 (33–44)	0.24
	HCO_3_	17 (14–19)	20 (19–21)	< 0.001 *
Prognosis	Improved	63 (63%)	100 (0%)	< 0.001 **
	Died	37 (37%)	0 (0%)	

*: Significant; CPAP: continuous positive airway pressure; MV: Mechanical ventilation; PH: power of hydrogen; PO2: partial pressure of oxygen; PCO2: partial pressure of carbon dioxide; HCO3: bicarbonate

The RDS group had lower median platelet count, PMI, and WBCs/MPV than the TTN and control groups and a lower PDW% and P-LCR% than the control group. RDS cases had a significantly higher MPV/platelet count compared to the TTN and control groups and a higher MPV compared to the TTN group only. Moreover, The TTN group had more WBCs, lymphocyte percentage, and count, but a lower neutrophil percentage than the RDS group (Table 3).

**Table 3:** WBCs profile and platelets indices in the studied groups

Variable median (IQR)	RDS (*N* = 100)	TTN (*N* = 100)	Controls (*N* = 100)	*P*-value
Platelets count (× 10^9^/L)	223 (192–279)	262 (201–297)	291 (211–396)	P1 < 0.001 ** P2 = 0.03 * P3 = 0.02 *
MPV (fl.)	8.6 (8.2–9.8)	8.5 (7.8–9.2)	8.5 (8.3–9.1)	P1 = 0.31 P2 = 0.09 P3 = 0.005 *
Platelets mass index (fL/nL)	1.923 (1.701–2.411)	2.114 (1.693–2.631)	2.444 (2.003–3.208)	P1 < 0.001 ** P2 = 0.003 * P3 = 0.09
MPV/platelets count	0.039 (0.032–0.047)	0.034 (0.028–0.042)	0.031 (0.025–0.041)	P1 < 0.001 ** P2 = 0.29 P3 = 0.003 *
WBCs/MPV	1090 (715–1448)	1455 (1129–1884)	1121 (839–1497)	P1 < 0.001 ** P2 < 0.001 ** P3 < 0.001 **
PDW (%)	14.1 (10.2–19.1)	16.7 (12.3–18.2)	17.9 (13.8–18.7)	P1 = 0.01 * P2 = 0.03 * P3 = 0.69
P-LCR (%)	33.6 (24–40.3)	32.5 (28.3–39.2)	37.8 (31.6–40.3)	P1 = 0.04 * P2 = 0.02 * P3 = 0.9
**WBCs profile**				
WBCs × 10^9^/L	10 (6.3–13.9)	12.6 (9.8–15.9)	10 (8.4–12.1)	P1 = 0.59 P2 < 0.001 ** P3 = 0.001 *
Monocytes (%)	4.5 (1.5–9.9)	4.8 (1–9.3)	3 (1.3–9.6)	P1 = 0.57 P2 = 0.45 P3 = 0.27
Monocytes count (x 10^9^/L)	290 (140–875)	649 (170–1110)	390 (135–1160)	P1 = 0.67 P2 = 0.62 P3 = 0.29
Neutrophils (%)	57.5 (46.7–73)	47.7 (32.3–57.1)	34.1 (24.7–59.1)	P1 < 0.001 * P2 = 0.16 P3 < 0.001 **
Neutrophils count (x 10^9^/L)	5.66 (3.7–8.3)	5.67 (3.76–8.1)	3.79 (2.24–6.56)	P1 = 0.001 * P2 = 0.002 * P3 = 0.79
Lymphocytes (%)	35 (17.6–48.4)	47.1 (30.5–62.7)	44.5 (19.4–68.8)	P1 = 0.004 * P2 = 0.96 P3 = 0.001 *
Lymphocyte count (x 10^9^/L)	2.86 (1.81–5.1)	5.1 (3.37–8.94)	4.49 (2.13–7.22)	P1 = 0.003 * P2 = 0.24 P3 < 0.001 **
Eosinophil (%)	0.6 (0.3–1.1)	0.5 (0.2–1.2)	0.5 (0.2–1.2)	P1 = 0.63 P2 = 1 P3 = 0.5
Eosinophil count (x 10^9^/L)	50 (13–190)	70 (20–170)	65 (10–160)	P1 = 0.45 P2 = 0.24 P3 = 0.22

*: Significant; P1: RDS vs. control groups; P2: TTN vs. control groups; P3: RDS vs. TTN groups; MPV: mean platelet Volume; PCT: platelet crit; PDW: platelet distribution width; P-LCR: platelet–large cell ratio; WBCs: white blood cells

The RDS group had significantly higher median serum LDH levels [(667 U/l (530–787)], NLR [1.9 (1–3.9)], and SII [353 (202–958)] than the TTN group where LDH was [500 U/l (420–660)], NLR [1.0 (0.5–1.8)], and SII [240 (133–454)], and the control group LDH [476 U/l (438–574)], NLR [0.8 (0.4–3)], and SII [190 (126–591)] with *p* < 0.001. The RDS group had a significantly higher median PLR [0.07 (0.04–0.11)] than the TTN group [0.05 (0.03–0.08)]. MLR was also higher in the RDS group [0.17 (0.03–0.4)] than in the TTN group [0.11 (0.02–0.25)]. The RDS group had a significantly higher median SIRI [884 (145–2126)] than the control group [650 (71–1653)]. The median P2/MS was considerably lower in the RDS group [204 (101–609)] than in the TTN [408 (128–1612)] and the control [416 (151–2630)] (Table 4).

**Table 4:** Systemic immune-inflammatory indices and LDH levels in the studied groups

Variable	RDS (*N* = 100)	TTN (*N* = 100)	Controls (*N* = 100)	*P*-value
NLR	1.9 (1–3.9)	1.0 (0.5–1.8)	0.8 (0.4–3)	P1 < 0.001 * P2 = 0.22 P3 < 0.001 *
MLR	0.17 (0.03–0.4)	0.11 (0.02–0.25)	0.13 (0.02–0.33)	P1 = 0.17 P2 = 0.324 P3 = 0.041*
LMR	5.9 (2.5–33.2)	9.4 (3.6–53.1)	7.7 (3.1–42.7)	P1 = 0.22 P2 = 0.35 P3 = 0.07
PLR	0.07 (0.04–0.11)	0.05 (0.03–0.08)	0.06 (0.03–0.12)	P1 = 0.16 P2 = 0.14 P3 = 0.004*
P2/MS	204 (101–609)	408 (128–1612)	416 (151–2630)	P1 < 0.001 * P2 = 0.35 P3 = 0.003 *
SII	353 (202–958)	240 (133–454)	190 (126–591)	P1 = 0.003 * P2 = 0.63 P3 = 0.004 *
SIRI	884 (145–2126)	677 (91–1713)	650 (71–1653)	P1 = 0.04 * P2 = 0.49 P3 = 0.2
EMR	0.59 (0.26–1.1)	0.46 (0.17–1.18)	0.46 (0.17–1.2)	P1 = 0.78 P2 = 0.48 P3 = 0.3
LDH (U/l)	667 (530–787)	500 (420–660)	476 (438–574)	P1 < 0.001 * P2 = 0.76 P3 < 0.001 *

*: significant; P1 = RDS vs. control groups; P2 = TTN vs. control groups; P3 = RDS vs. TTN groups; NLR: neutrophil lymphocyte ratio; MLR: monocyte lymphocyte ratio; LMR: lymphocyte monocyte ratio; PLR: platelet lymphocyte ratio; p2: (platelet count)2; MS: monocyte fraction × segmented neutrophil fraction; SII: systemic immune inflammation index; SIRI: systemic inflammation response index; EMR; eosinophil monocyte ratio

**Fig. 1:**
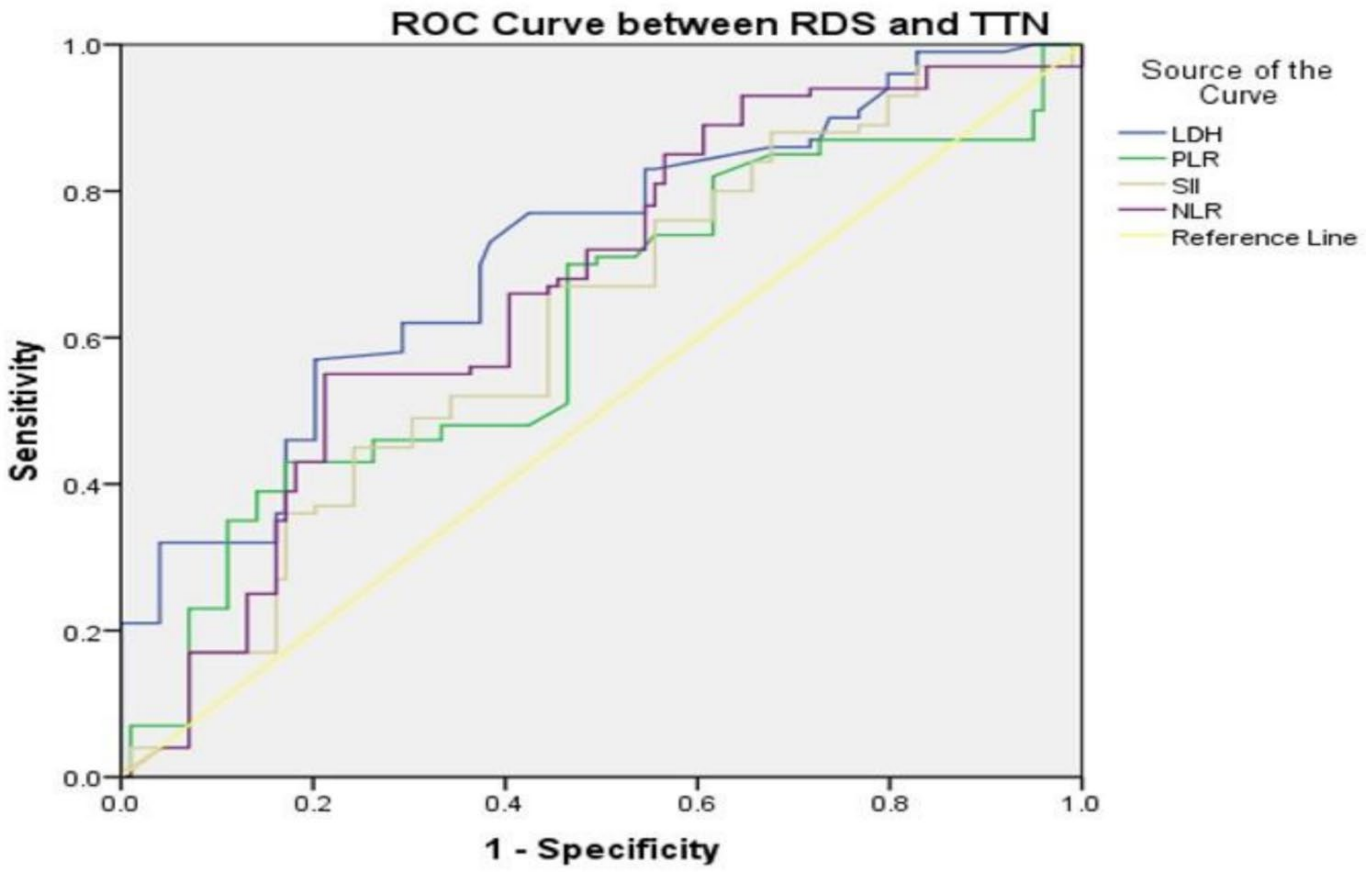
ROC curve of laboratory markers distinguishing between RDS and TTN groups

ROC curve analysis revealed that to discriminate between the RDS and TTN groups, serum LDH at a cut-off level of > 660 U/L had an AUC of 0.719 with 57% sensitivity, 80% specificity, 74% PPV, and 65% NPV, followed by NLR at a cut-off level of > 0.90 with an AUC of 0.667 with 85% sensitivity, 43% specificity, 59% PPV, and 73% NPV. followed by PLR at a cut-off level of > 0.085 with an AUC of 0.619, which had 43% sensitivity, 83% specificity, 71.1% PPV, and 59.3% NPV, whereas SII at a cut-off level of > 245.57 with an AUC of 0.619, which had 67% sensitivity, 56% specificity, 60.4% PPV, and 62.9% NPV (Fig. 1).

**Fig. 2:**
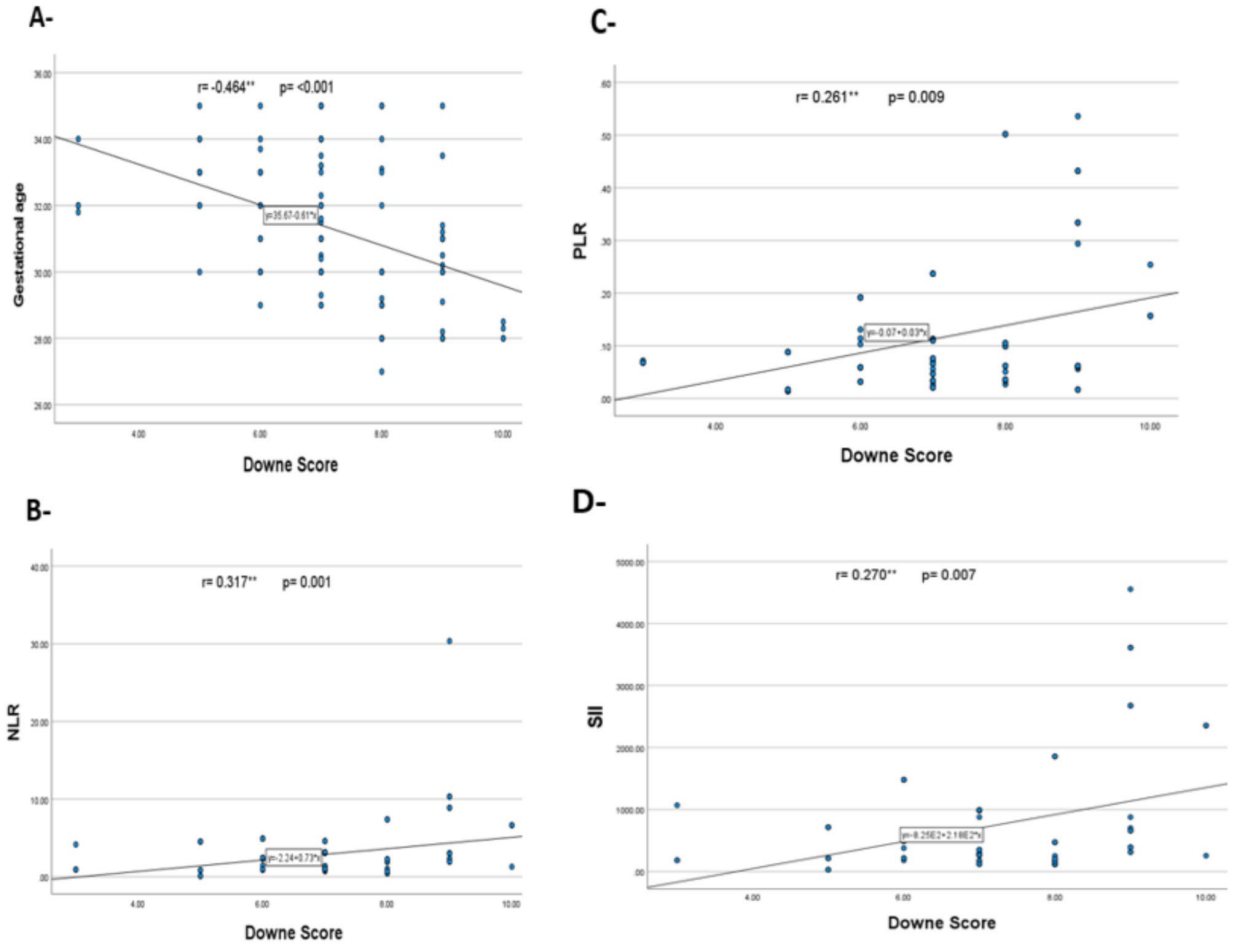
Correlation analysis between Downe score with (**A**) gestational age, (**B**) neutrophil-lymphocyte ratio (NLR), (**C**) platelet-lymphocyte ratio (PLR), and (**D**) Systemic immune-inflammation index (SII) in neonates with respiratory distress syndrome

In neonates with RDS, A significant negative correlation was detected between the Downe score and gestational age (*r* = − 0.464**, *p* = < 0.001), while significant positive correlations were detected with PLR (*r* = 0.261**, *p* = 0.009), NLR (*r* = 0.317**, *p* = 0.001), and SII (*r* = 0.270**, *p* = 0.007) as shown in Fig. 2.

**Fig. 3:**
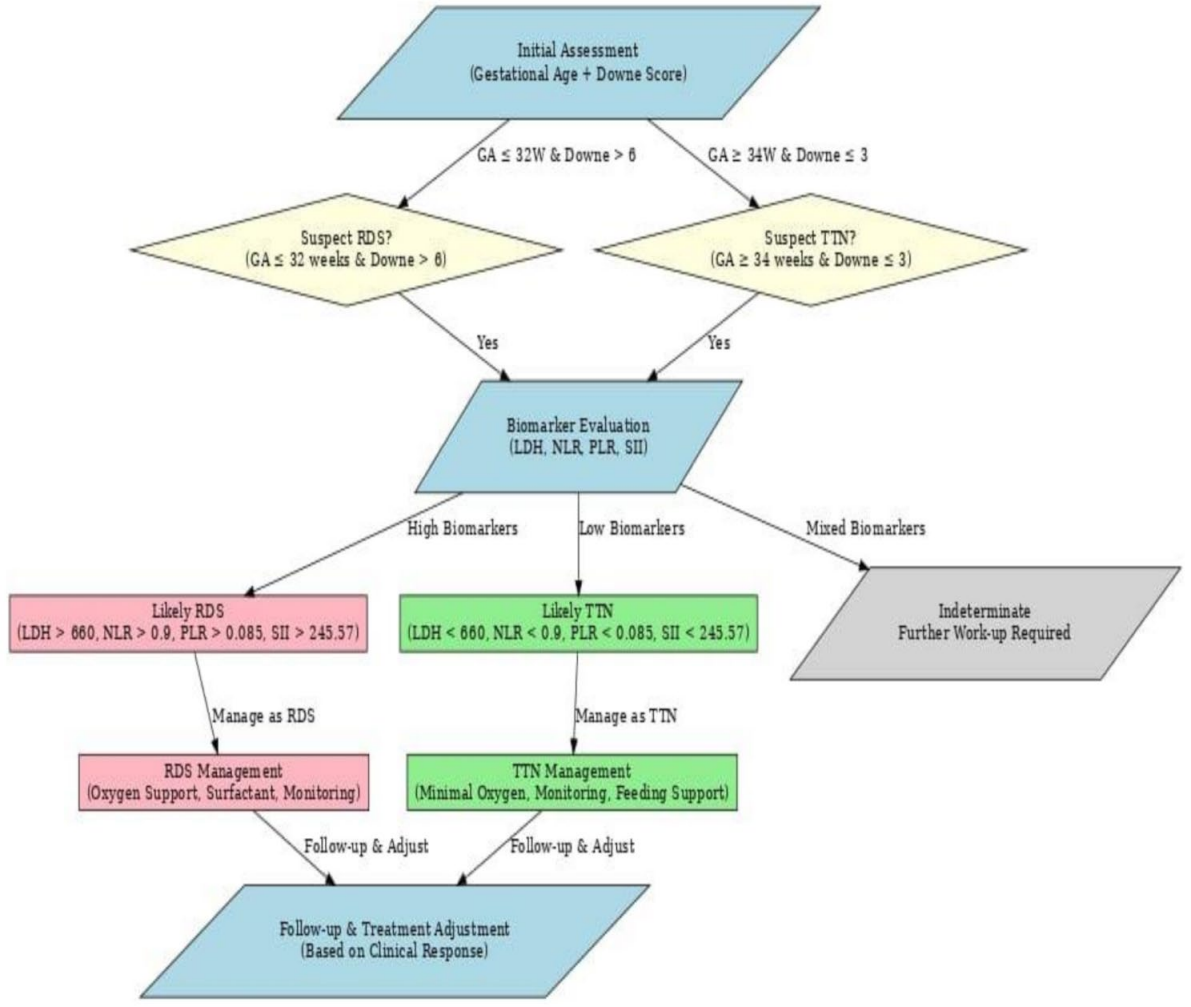
Integrated Biomarker-Based Algorithm for Differentiating Neonatal Respiratory Distress syndrome and transient tachypnea of the newborn

Figure 3 shows an algorithm that can help differentiate RDS from TTN in neonates with respiratory distress by integrating biomarkers with clinical signs. Combining the marker with key clinical indicators (e.g., Downe score, gestational age) can provide a more comprehensive and accurate approach to diagnosing and managing respiratory distress cases.

## Discussion

Early differentiation between TTN and RDS is important to guide appropriate treatment decisions. While TTN often resolves spontaneously with supportive care, RDS may necessitate interventions, such as surfactant replacement therapy and mechanical ventilation ((33–34).

In the current study while TTN cases had a median gestational age of 33 weeks with 38% being LGA, neonates with RDS had a median gestational age of 31 weeks with 18% being small for gestational age and 71% being AGA. According to previous reports, prematurity is the most significant risk factor for RDS ((35–36). Jensen et al. [37] reported that in low-income countries, respiratory distress syndrome (RDS) is the primary cause of death for premature neonates. Several studies demonstrating that LGA neonates are at increased risk for developing TTN ((33, 38–39), while the prevalence of being SGA among neonates with respiratory distress syndrome varies across studies. In a cohort of 515 preterm infants born before 30 weeks gestational age, 19% were identified as SGA. This study found that while SGA status was significantly associated with an increased risk of bronchopulmonary dysplasia (BPD), it was not linked to a higher incidence of RDS or mortality [40]. Conversely, a study analyzing 883 infants born before 33 weeks gestation found that 27.6% were SGA. In this cohort, SGA infants born between 25 and 28 weeks gestation had an increased incidence of RDS compared to AGA infants, while at gestational age 29–32 weeks, SGA-infants had a lower incidence of RDS as compared to AGA-infants [41]. These variations highlight the complexity of the relationship between SGA status and respiratory outcomes in preterm infants.

Blood gases are critical in diagnosing and monitoring respiratory disorders in newborns, particularly RDS cases, for follow-up on respiratory support, whether invasive or non-invasive ventilation [42]. Our study found that RDS neonates had lower pH, PO2, and HCO3 levels than the TTN group, indicating poorer oxygenation and a higher degree of acidosis. We also found that RDS patients required more extensive oxygen therapy and had a longer hospital stay than TTN patients, with a worse prognosis and greater fatality rates. Consistent with our findings, the study by Panigrahi et al. [43], highlighted the widespread use of oxygen support in neonates with respiratory distress, indicative of the critical nature of this condition. Elfarargy et al. [44], reported significant differences in blood pH among neonates in the RDS, TTN, and control groups, with the RDS group demonstrating the lowest pH levels. Another study by Liu and Tong [45] showed that RDS neonates exhibited significantly lower pH levels than TTN neonates, indicating a greater degree of acidosis during the early postnatal period. However, significantly lower PaO2 levels were observed in the TTN group, suggesting impaired oxygenation and respiratory function in neonates with TTN.

In agreement with our results as regards LDH levels, Lee et al. [46], found that both the RDS and TTN groups had increased LDH levels upon admission, especially in the RDS group compared to the TTN group. They also found significant correlations between LDH levels and the duration of respiratory support required and hospital stay in both groups. This might represent the extent of continuing damage and the prognosis of infants experiencing respiratory distress.

Additionally, the study by Van Anh et al. [47], showed that infants requiring CPAP for respiratory distress had significantly higher LDH levels than those who did not require CPAP. This suggests that LDH levels may serve as a valuable biomarker for assessing the severity and prognosis of respiratory distress in neonates, particularly for predicting the need for respiratory support and hospitalization duration. Aydogdu et al. [48] also reported that serum enzymes, including LDH, were elevated in patients with RDS without preceding perinatal hypoxia. This finding suggests that LDH could serve as a marker for primary lung injury in RDS.

Furthermore, Ozkiraz et al. [2] proposed that an initial LDH level with a cut-off value of 750 U/L may predict prolonged oxygen requirements in patients with TTN. This indicates the prognostic value of LDH in identifying newborns at risk of respiratory deterioration, emphasizing its potential utility in the clinical management and risk assessment of TTN. Lim and Kim [49] reported a negative correlation between LDH levels and oxygenation during ventilator-induced lung damage.

Infection, artificial ventilation, and high oxygen concentrations can disrupt the normal development of immature lungs [50], causing a sequence of inflammatory reactions that can lead to acute and chronic lung injury in newborn children [51]. Our study identified distinct hematological profiles in neonates with RDS, characterized by altered platelet dynamics, and heightened inflammatory responses compared to TTN and controls.

In addition to changing the function of type II epithelial cells, inflammatory mediators can also change the synthesis, secretion, and recycling of pulmonary surfactants. Thus, it is critical to forecast the course of NRDS using systemic inflammatory markers in order to avoid and improve the prognosis in advance. Weng et al., [52] in their study revealed that maternal systemic inflammatory indices were independent risk factors for NRDS, and their combined predictive efficacy is better. Another study by Yuce [53] revealed that among mothers NLR and PLR values predict spontaneous preterm birth with high sensitivity and specificity.

Bolat et al. [54] found that NLR both at birth and at 72 h of life can be used as a biomarker to distinguish TTN patients from healthy newborns and to predict TTN severity, suggesting potential immune system involvement in TTN pathophysiology. Other studies have shown that neutrophil abnormalities, lymphocyte abnormalities, or platelet abnormalities can lead to alveolar injury. The study by Wang et al. [55], and Mısırlıoğlu et al. [56], concluded that the NLR is a valuable biomarker for predicting and assessing the severity of acute respiratory distress syndrome (ARDS). They also suggested that NLR can be a convenient and inexpensive parameter for diagnosing the severity of ARDS, particularly in cases of severe acute hypoxemic respiratory insufficiency.

In a recent study by Cakir et al. [57], premature infants with RDS had significantly higher NLR, PLR, and SII values than those in the non-RDS group. In terms of the predictivity of RDS, SII had a cut-off value of ≥ 78.200 with an AUC of 0.842. Multiple logistic regression analysis showed that a higher SII level (≥ 78.2) was independently associated with RDS.

In line with our findings, a decreased platelet count and PMI were significantly associated with TTN compared to the control group [58]. Canpolat et al. [59] found that MPV was considerably higher in RDS patients than in the non-RDS group, related to young platelet production as a result of increased platelet consumption in pulmonary damage due to RDS.

Our study had multiple strengths as potential biases in patient selection were addressed by following clear and specific inclusion and exclusion criteria. Our study also had a prospective deign where patients were categorized in real-time, reducing selection bias that may occur in retrospective studies. Also comparing our data with a healthy control group verifying that the observed outcomes are not influenced by factors unrelated to our study aims. However, the study’s dependence on a small sample size is one of its limitations, which could have an impact on how broadly the results can be applied. The conclusions and results’ robustness would be enhanced by a bigger, more varied sample size. Another drawback of our study, along with the absence of long-term follow-up of the patients, is the challenge of enrolling healthy preterm neonates as a control group while choosing a healthy full-term group. The usefulness of these markers and the improvement of their diagnostic thresholds may be confirmed by external validation studies conducted in bigger, diverse populations.

## Conclusion

Newborns with RDS have more tissue damage, cell turnover, and an increased inflammatory response than TTN and controls. Even with moderate sensitivity and specificity, LDH levels, platelet indices, and inflammatory indices can be helpful for early triage in addition to the clinical evaluation of the cases and blood gases analysis. These markers are affordable, widely accessible, and easy to measure. Combined use of platelet and systemic inflammatory indices can improve clinical outcomes by providing a quick and affordable way to differentiate between disorders like RDS and TTN for improving clinical outcomes.

## Data Availability

The datasets used and/or analyzed during the current study are available from the corresponding author upon reasonable request, after obtaining the permission of our institute.

## Declarations

### Clinical trial number

SVUMEDPED0254245850.
